# Ovine congenital progressive muscular dystrophy (OCPMD) is a model of TNNT1 congenital myopathy

**DOI:** 10.1186/s40478-020-01017-1

**Published:** 2020-08-20

**Authors:** Joshua S. Clayton, Elyshia L. McNamara, Hayley Goullee, Stefan Conijn, Keren Muthsam, Gabrielle C. Musk, David Coote, James Kijas, Alison C. Testa, Rhonda L. Taylor, Amanda J. O’Hara, David Groth, Coen Ottenheijm, Gianina Ravenscroft, Nigel G. Laing, Kristen J. Nowak

**Affiliations:** 1 Harry Perkins Institute of Medical Research, Queen Elizabeth II Medical Centre, Nedlands, 6009 WA Australia; 2 Centre for Medical Research, Queen Elizabeth II Medical Centre, University of Western Australia, Nedlands, 6009 WA Australia; 3 Department of Physiology, Amsterdam University Medical Center (Location VUmc), Amsterdam, Netherlands; 4 Animal Care Services, University of Western Australia, Nedlands, 6009 WA Australia; 5 Commonwealth Scientific and Industrial Research Organisation Agriculture and Food, Queensland Bioscience Precinct, Brisbane, 4067 QLD Australia; 6 Faculty of Health and Medical Sciences, School of Biomedical Sciences, Queen Elizabeth II Medical Centre, University of Western Australia, Nedlands, 6009 WA Australia; 7 School of Veterinary Medicine, Murdoch University, Murdoch, 6150 WA Australia; 8 School of Pharmacy and Biomedical Sciences, CHIRI Biosciences Research Precinct, Curtin University, Bentley, 6102 WA Australia; 9 Office of Population Health Genomics, Public and Aboriginal Health Division, Western Australian Department of Health, East Perth, 6004 WA Australia

**Keywords:** Congenital myopathy, Sheep model, Troponin T1, TNNT1, Splicing, Ca^2+^ sensitivity, Neuromuscular disease, Skeletal muscle, OCPMD, Muscular dystrophy, Nemaline myopathy

## Abstract

Ovine congenital progressive muscular dystrophy (OCPMD) was first described in Merino sheep flocks in Queensland and Western Australia in the 1960s and 1970s. The most prominent feature of the disease is a distinctive gait with stiffness of the hind limbs that can be seen as early as 3 weeks after birth. The disease is progressive. Histopathological examination had revealed dystrophic changes specifically in type I (slow) myofibres, while electron microscopy had demonstrated abundant nemaline bodies. Therefore, it was never certain whether the disease was a dystrophy or a congenital myopathy with dystrophic features. In this study, we performed whole genome sequencing of OCPMD sheep and identified a single base deletion at the splice donor site (+ 1) of intron 13 in the type I myofibre-specific *TNNT1* gene (KT218690 c.614 + 1delG). All affected sheep were homozygous for this variant. Examination of *TNNT1* splicing by RT-PCR showed intron retention and premature termination, which disrupts the highly conserved 14 amino acid C-terminus. The variant did not reduce TNNT1 protein levels or affect its localization but impaired its ability to modulate muscle contraction in response to Ca^2+^ levels. Identification of the causative variant in *TNNT1* finally clarifies that the OCPMD sheep is in fact a large animal model of TNNT1 congenital myopathy. This model could now be used for testing molecular or gene therapies.

**Electronic supplementary material:**

The online version of this article (10.1186/s40478-020-01017-1) contains supplementary material, which is available to authorized users.

## Introduction

Ovine congenital progressive muscular dystrophy (OCPMD) was first described in a Merino sheep flock in Queensland, Australia in 1969 [1]. Further cases were subsequently characterized in Merino sheep in Western Australia in 1979 [2]. Although the clinical and pathological features of both flocks are highly similar, there is no known breeding connection between these flocks [2]. Herein, we will use the term OCPMD to describe the Western Australian flock. The most prominent feature of OCPMD is a distinctive gait with stiffness and reduced flexion of the hind limbs that is more pronounced after exercise [3]. The first signs of the disease are observed within 1 month after birth [3]. Many affected sheep develop a bloated, pot-bellied appearance at 9–12 months of age and some exhibit respiratory distress following mild exercise [2]. Most affected animals are either culled or die of other causes before 2 years of age [2]. Affected sheep exhibited higher resting and post-exercise creatine kinase and lactate dehydrogenase levels [3]. Affected muscles were type I (slow/oxidative) myofibre-predominant [3]. As a progressive disorder, skeletal muscle wasting continues throughout life and skeletal muscles, especially those with high proportions of type I myofibres, are gradually replaced by fat [3]. Histopathology [4] and myofibre morphometry [5] indicated confinement of OCPMD pathology to type I myofibres. Electron microscopy showed large numbers of nemaline bodies in the affected myofibres [6].

OCPMD has a recessive inheritance pattern [7]. Given that type I myofibres are selectively affected, we hypothesized that the causative mutation would be within a gene coding for a protein expressed in type I skeletal myofibres. We previously used protein analysis methods to assess targets with known involvement in skeletal muscle disease ([8] and unpublished data), but these failed to yield any strong candidates. Here, using whole genome sequencing (WGS), we identified a single base deletion within the essential splice donor site of intron 13 of the *TNNT1* gene, which codes for the type I myofibre-specific troponin T protein (TNNT1). Using functional analyses we investigated the mechanism by which the variant causes disease, and clarify OCPMD as a model of TNNT1 congenital myopathy.

## Materials and methods

### Phenotypic characterisation

Affected animals and carriers were all derived from the original Western Australian flock of OCPMD sheep [2]. Disease status was scored based upon the following criteria: (1) noticeably stiffened gait in the hind limbs, leading to reduced ability to run effectively (in comparison to unaffected individuals), (2) skeletal muscle tissue wasting in affected muscle groups with adipose replacement, (3) presence of nemaline bodies in affected muscle tissues at biopsy, and (4) raised creatine kinase at rest, and particularly after exercise (in comparison to unaffected individuals). ‘Affected’ individuals could be descended from: (1) two affected individuals (100% chance), (2) an affected and a carrier, along with phenotypic presentation of disease pathology (50% chance), or (3) from two known carrier individuals, along with phenotypic presentation of disease pathology (25% chance). ‘Carrier’ individuals were descendants of either an affected and a carrier (50% chance), or from an affected and an unaffected individual (100% chance). ‘Wild-type’ individuals did not show any clinical signs of disease and were descended either from two animals outside the directed pedigree (close to 100% chance) or from two carrier individuals (25% chance). A full pedigree of the sheep flock analysed in the present study may be found in Additional File 1: Fig S1.

### Animal husbandry and care

Sheep were housed outdoors in 0.5–1.0 acre paddocks exposed to ambient weather, with natural shelter (trees). Wild-type, carrier and affected sheep were all housed together in small sex-specific groups of 2–15 sheep. Sheep were maintained on a weighed ration of commercial ewe and lamb pellets (17.2% crude protein) along with oaten chaff and a loose sheep mineral mix. For pregnant and lactating ewes, mineral mix was supplemented with di-calcium phosphate. They also had access to a variety of grass and legume species in the paddock. Oaten hay was provided during times of low pasture availability, and Lucerne hay was provided to late pregnant ewes, at lambing, and until weaning time.

Sheep were maintained using industry best practice procedures. Lambs were vaccinated against Caseous Lymphadenitis and five clostridial diseases at 4 and 8 weeks of age and then annually (Glanvac 6, Zoetis Australia Pty Ltd); contagious ecthyma once as lambs (Scabigard, Zoetis Australia Pty Ltd); and Ovine Johne’s Disease once as lambs (Gudair, Zoetis Australia Pty Ltd). Individual faecal egg counts were performed at regular intervals throughout the year, and individual sheep were administered an anthelmintic (rotating drugs) if indicated by a high egg count. A preventative off-shears lice treatment (Spinosad, Extinosad Pour On, Elanco, Australia) was applied at shearing time. The sheep were weighed monthly and body condition scored on a weekly basis. Low stress sheep handling techniques were used and long-distance walks during hot weather were avoided.

Breeding was performed either by timed mating with natural sires, or by artificial insemination and embryo transfer (Genstock Pty Ltd, Kojonup, Western Australia). Oestrous cycle in ewes was synchronized using a controlled internal drug release device. Rams used for natural mating wore breeding harnesses in order to record mating dates. Affected rams had no apparent issues with natural mating, although breeding groups were housed in small yards to reduce the distance to ewes, and the ratio of ewes to rams was 4:1. Ewe lambs were not naturally mated until at least 12 months old. One month prior to expected lambing date, ewes were moved to a paddock with additional shelter. As lambs were born, the ewe and lamb were placed in individual temporary pens (lambing ‘jugs’) for 2–7 days to ensure good maternal behaviour and to allow close monitoring. Lambs were weighed and tagged 1–2 days after birth for identification purposes and released with the ewe into the paddock. Lambs had access to supplemental feed (alongside their mothers) from day one. Lambs were weaned at 3–4 months old, at which point they were weighed weekly for 1–2 months to ensure good growth.

### SNP array and linkage analysis

Genomic DNA from 14 affected sheep and 6 carriers were analysed on an OvineSNP50 BeadChip (Illumina, USA). Typed SNPs that passed quality control standards were output in PLINK format (.ped) alongside a genomic map position of each SNP (.map). As existing homozygosity callers function off human datasets, homozygosity mapping was performed using a custom perl script. Homozygous region runs that were present only in carriers were discounted from further analyses. Runs shared between carriers and affected sheep were considered as potential candidates but were given less weight than runs present only in affected individuals. Association mapping was performed using PLINK [9], although the power of these tests was limited due to the small sample size, relatively sparse genome coverage and the inability to test relatedness of individuals. Linkage analysis was performed using the Merlin software package [10].

### Whole genome sequencing

Illumina whole genome sequencing was performed on two affected sheep at 50× coverage by the service provider (Broad Institute). Reads were aligned to the *Ovis aries* (Texel sheep) reference genome, version 3.1 (Oar v3.1) [11] and variants called using the Genome Analysis Toolkit (GATK v3.5) [12]. Homozygous variants were extracted from each variant call format (vcf) file, and hard filtering used to exclude low quality calls (quality of depth, QD > 20; depth, DP > 10). A subset of around 4 million variants common to both affected sheep was extracted and annotated using Ensembl Variant Effect Predictor (VEP, release 85).

Variants were subsequently filtered to include only those within exons or introns of known protein-coding genes. Low coverage (5×) Proton™ WGS data from an additional four affected and eight unaffected, unrelated Merino sheep (wild-type “controls”) were used to narrow down the list of potential disease-causing variants. As poor coverage limited accuracy and zygosity calling, these data were used only to support or refute the potential pathogenicity of high-quality homozygous variants identified in the two Illumina WGS datasets. A pipeline consisting of samtools [13], vt [14], and in-house python scripts was used to perform these analyses.

### RT-PCR and DNA sequencing

To extract RNA, 20–30 µg cryopreserved sheep skeletal muscle tissue was homogenized in 300 µL Buffer RLT using a mini-bead beater (BioSpec Products, USA) at maximum oscillations per min in 30 s intervals. Total RNA was isolated using a RNeasy fibrous tissue mini kit with on-column DNase I digestion (QIAGEN, USA) and cDNA generated using the SuperScript III first-strand synthesis system (Thermo Fisher, USA). Standard RT-PCR reactions (25 µL) comprised: 1× GoTaq G2 master mix (Promega, USA), 0.5 µM forward and reverse primer, and 1 µL diluted cDNA.

Typical thermocycling conditions were as follows: 95 °C for 5 min; 15 cycles of touchdown PCR: 95 °C for 30 s, 65 °C for 30 s (− 0.5 °C/cycle), 72 °C for 2 min; 25 cycles of: 95 °C for 30 s, 57 °C for 30 s, 72 °C for 2 min, and a final extension at 72 °C for 5 min. Full-length *TNNT1* cDNA was amplified using Platinum SuperFi PCR master mix (Thermo Fisher, USA). Cycling was as follows: 98 °C for 30 s, 35 cycles of 98 °C for 10 s, 60 °C for 10 s and 72 °C for 2 min, and a final extension at 72 °C for 5 min. A full list of primers may be found in Additional file 2: Table S1. PCR products were analysed on a 1% agarose gel and fragments of interest purified using a QIAquick gel extraction kit (QIAGEN, USA). Purified products were sequenced by the Sanger method [15] and chromatograms aligned to reference transcripts in Benchling (benchling.com) using the MAFFT algorithm [16, 17].

### TA cloning of RT-PCR products

As direct sequencing of some PCR products was unsuccessful or did not allow sequencing of the entire amplicon, PCR reactions were cloned into the pCR-2.1 vector using a TOPO TA cloning kit (Thermo Fisher, USA). Individual clones were picked and plasmid DNA isolated using a QIAprep spin miniprep kit (QIAGEN, USA). Plasmids were then screened for insert presence by digestion with EcoRI-HF (NEB, USA). Cloned PCR products were sequenced in both directions using M13 forward and M13 reverse primers (Additional file 2: Table S1) and aligned to the published Dorper sheep *TNNT1* cDNA sequence (Accession #KT218690) [18] in Benchling (benchling.com) using the MAFFT algorithm [16, 17].

### Protein multiple sequence alignments

TNNT1 amino acid sequences from sheep and six other mammalian species were aligned using Clustal Omega v1.2.4 [19]. Protein sequences used for alignments were as follows: sheep (*Ovis aries*): AMR55385 (published AA sequence from K218690 CDS [18]), human (*Homo sapiens*): NP_0011196044 (NCBI RefSeq), cow (*Bos taurus*): NP_776899 (NCBI RefSeq), mouse (*Mus musculus*): NP_001264833 (NCBI RefSeq), rat (*Rattus norvegicus*): NP_001264191 (NCBI RefSeq), and dog (*Canis lupus familiaris*): XP_005616225 (NCBI predicted).

### Western blot analysis

Total protein extracts were isolated from vastus intermedius muscle samples by homogenisation in lysis buffer (8 M urea, 125 mM Tris, 40% glycerol, 4% SDS; pH 8.8; and 15% protein inhibitor cocktail IV). Samples were heated at 95 °C, centrifuged for 5 min at 10,000 *g* and the supernatant collected. Total protein levels were measured using the bicinchoninic acid detection kit (Thermo Fisher, USA). Protein samples (10 µg) were briefly sonicated for 1 min before being resolved on 4–12% bis-tris midi gels (Thermo Fisher, USA), transferred to a polyvinylidene difluoride membrane (Thermo Fisher, USA) at 300 mA for 2 h at room temperature, and blocked for 1 h in 5% skim milk powder in PBS and 0.1% Tween 20. Membranes were incubated with a primary antibody against TNNT1 (1:5000; Sigma, Australia; HPA058448) at 4 °C overnight. Suitable horseradish peroxidase-conjugated secondary antibodies were used for detection with the Pierce™ ECL plus western blotting substrate kit (Thermo Fisher, USA). The gel was post-stained with Coomassie blue and the myosin band used as a loading control.

### Histology and immunohistochemistry

Skeletal muscle samples were frozen in liquid nitrogen-cooled 2-methylbutane and stored at − 80 °C. Serial 10 µM sections were cut on a Leica CM3050S cryostat and used for standard histochemical techniques. For Gomori trichrome staining, sections were incubated in Gill’s haematoxylin (Sigma, Australia) for 7 min, washed with tap water, and then incubated with Gomori trichrome stain for 6 min. Slides were rinsed with 1% acetic acid, washed twice in 100% ethanol, washed twice in xylene substitute, and mounted in Entellan (ProSciTech, Australia).

Serial 10 µM muscle sections were blocked in phosphate buffered saline (PBS) with 10% fetal calf serum (FCS; Gibco, USA) and 1% bovine serum albumin (BSA; Sigma, Australia) for 1 h at room temperature. Rabbit polyclonal TNNT1 antibody (diluted 1:30; Sigma, Australia; HPA058448) or mouse monoclonal alpha-actinin antibody (diluted 1:20; Sigma, Australia; Clone EA-53; A7732) was applied and incubated at 4 °C overnight. Slides were washed in PBS, then incubated with phalloidin tetramethylrhodamine (diluted 1:1000; Sigma, Australia; P1951) and suitable secondary antibodies (diluted 1:500; Life Technologies, USA) for 1 h at room temperature. Lastly, slides were washed in PBS, counterstained in Hoechst (Sigma, Australia) and mounted in Hydromount (Electron Microscopy Sciences, USA).

All sections were imaged on a fluorescence microscope (model IX-71 or BX51, Olympus) equipped with a digital camera (model DP-74 or DP-80, Olympus).

### Permeabilized myofibre mechanics

Skeletal muscle specimens were thawed and small strips were dissected from the muscle biopsies and permeabilized overnight as described previously [20, 21]. This procedure permeabilizes membranous structures in the myofibres, which enables activation of the myofilaments with exogenous calcium. Preparations were washed thoroughly with relaxing solution and stored in 50% glycerol/relaxing solution at − 20 °C. Single myofibres were dissected from the permeabilized strips and mounted using aluminium T-clips between a length motor (ASI 315C-I, Aurora Scientific Inc., Canada) and a force transducer element (ASI 403A, Aurora Scientific Inc., Canada) in a single myofibre apparatus (ASI 802D, Aurora Scientific Inc., Canada) mounted on the stage of an inverted microscope (Zeiss Axio Observer A1). Sarcomere length was determined using a high-speed VSL camera and ASI 900B software (Aurora Scientific Inc., Canada). Mechanical experiments were performed at a sarcomere length of 2.5 μm to ensure that the sarcomeres operate at an optimal length (middle of the plateau phase). Myofibre width and diameter were measured at three points along the fibre and the cross-sectional area was determined assuming an elliptical cross-section. The bathing solutions used during the experimental protocols were: a relaxing solution (100 mM BES, 6.97 mM EGTA, 6.48 mM MgCl_2_, 5.89 mM Na_2_-ATP, 40.76 mM K-propionate, 14.5 mM creatine phosphate), a pre-activating solution with low EGTA concentration (100 mM BES, 0.1 mM EGTA, 6.42 mM MgCl_2_, 5.87 mM Na_2_-ATP, 41.14 mM K-propionate, 14.5 mM creatine phosphate, 6.9 mM HDTA), and an activating solution (100 mM BES, 7.0 mM Ca-EGTA, 6.28 mM MgCl_2_, 5.97 mM Na_2_-ATP, 40.64 mM K-propionate, 14.5 mM creatine phosphate). The temperature of the bathing solutions was kept constant at 20 °C using a thermocouple thermometer/TEC controller (ASI 825A, Aurora Scientific Inc., Canada).

To investigate submaximal force generating capacities at the sarcomere level, force-pCa relations were established. To determine the force-pCa relation (pCa = − log of molar free Ca^2+^ concentration), permeabilized myofibres were sequentially bathed in solutions with pCa values ranging from 4.5 to 9.0 and the steady-state force was measured. Force values were normalized to the maximal force obtained at pCa 4.5. The obtained force-pCa data were fit to the Hill equation, providing the pCa_10,20,50_ and the Hill coefficient, n_H_, an index of myofilament cooperativity. Statistical differences between matched wild-type and affected samples were assessed using a two-tailed, unpaired t-test.

### Myosin heavy chain isoform composition

As the contractile properties of myofibres are influenced by their myosin heavy chain composition, we used a specialized SDS-PAGE technique to analyse the myosin heavy chain isoform composition in the myofibres used in contractility experiments [20]. In brief, skeletal muscle fibres were denatured by boiling for 2 min in SDS sample buffer. The stacking gel contained a 4% acrylamide concentration (pH 6.7), and the separating gel contained 7% acrylamide (pH 8.7) with 30% glycerol (v/v). The gels were run for 24 h at 15 °C and a constant voltage of 275 volts. Gels were silver-stained, scanned, and analysed with ImageQuant TL software (GE Healthcare, USA).

### Graphs and statistics

Graphs and statistics were generated using GraphPad Prism V8. Sample comparisons were performed using a two-tailed, unpaired t-test. Statistical significance was assigned based on *p* < 0.05. Error bars represent mean ± SD.

## Results

### Gross observation and histochemical analysis confirmed type I (slow) myofibres are predominantly affected in OCPMD sheep

The skeletal muscle histopathology and myofibre morphometry of OCPMD sheep was characterized in detail in the 1970s and 1980s [2, 4, 5]. Here, we provide a brief update and confirm that the original disease features are present in the modern-day flock.

**Fig. 1:**
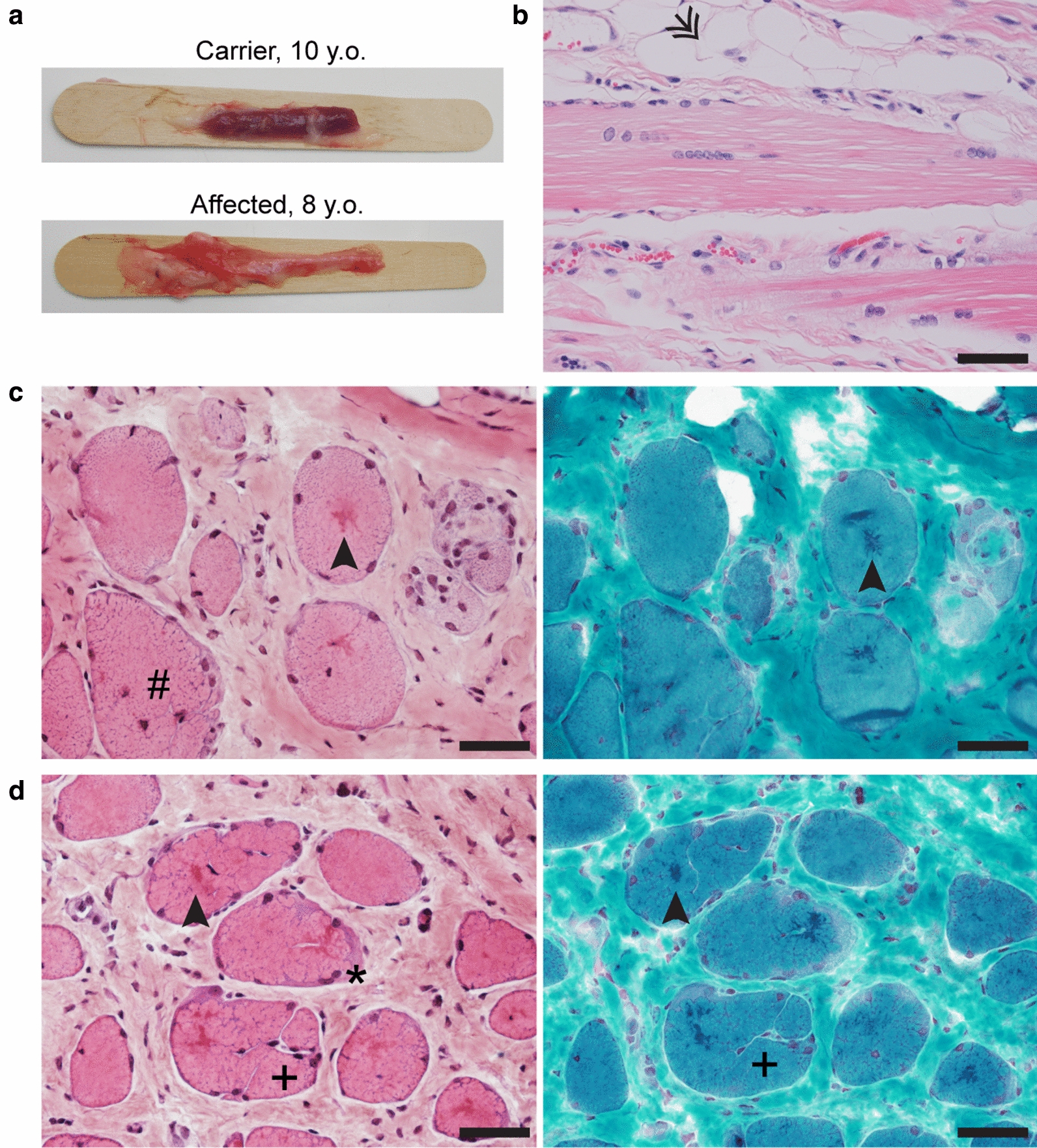
Type I-predominant skeletal muscles from OCPMD-affected sheep show extensive muscle degeneration and likely nemaline bodies. **a** Whole soleus muscles from a 10-year old unaffected carrier ewe and an 8-year old affected ewe. **b** Hematoxylin and eosin (H&E) stained longitudinal section of type I myofibre-predominant skeletal muscle (soleus) from an OCPMD-affected sheep; shows increased presence of collagenous connective tissue and fat (white/unstained; e.g. area indicated by arrow). **c**, **d** H&E (left) and Gomori trichrome (right) staining of soleus muscles from 5-year old (**c**) and 8-year old (**d**) affected sheep. Shows extensive variation in myofibre size, central nuclei (#), split myofibres (+), and basophilic infiltration (*). Gomori stains show the presence of sarcomeric protein aggregates—likely nemaline bodies within myofibres (dark areas, see arrowheads). All scale bars = 50 µm

At the gross level, type I myofibre-predominant muscles were always affected (sternocephalicus, vastus intermedius, soleus, anconeus, triceps) while other muscles were not (flexor carpi radialis, sartorius, semi-membranosus, and gracilis). Macroscopic examination of the vastus intermedius, anconeus and medial triceps found these muscles were generally flat, pale pink or had white streaking, and were firm to cut. Compared to those from age-matched carrier sheep, affected soleus muscles were markedly reduced in size and contained bands of fatty white tissue (Fig. 1a).

Histopathology of OCPMD-affected muscles confirmed the presence of mild to marked individual and grouped myofibre degeneration, as well as necrosis and regeneration with scattered subsarcolemmal vacuolation, myofibre hypertrophy, occasional split myofibres, and extensive replacement by fibro-adipose connective tissue (Fig. 1b). There was also extensive variation in myofibre diameter, and many myofibres were swollen or degenerating and/or contained centrally located nuclei (Fig. 1c,d). Additionally, many type I myofibres contained central sarcomeric protein aggregates (Fig. 1c,d). Thus, the pathology is consistent with both myopathic and dystrophic processes. Type II (fast) myofibre-predominant or mixed myofibre-type muscles showed little to no evidence of a dystrophic phenotype.

OCPMD is a slowly progressive disorder. Accordingly, comparison of type I myofibre-predominant muscles from wild-type, carrier and affected sheep of different ages confirm muscle degeneration at 1 year and extensive degeneration by 8 years of age in affected sheep (Additional file 3: Fig. S2). We note that the most severely affected muscles (vastus intermedius, soleus and anconeus) are comprised of 100% type I myofibres in wild-type and affected sheep of various ages [5]. Based on breeding and segregation the disease is recessively inherited. However, evidence of mild pathology typical of OCPMD-affected muscles was found in post mortem analysis of one 10-year old carrier, suggesting the causative gene/mutation may produce some pathological features in carriers.

### Whole genome sequencing identified a homozygous single nucleotide deletion at the splice donor site in intron 13 of the *TNNT1* gene in affected sheep

The sheep (*Ovis aries*) genome build version 3.1 was released in 2010 [11] and expanded the possibilities of genetic studies in sheep. To identify potential linkage regions for OCPMD, we used a single nucleotide polymorphism (SNP) array of > 54,000 variants approximately spread evenly throughout the sheep genome (as annotated from the v3.1 reference). Homozygosity and association mapping did not identify a linkage region although these analyses were limited by the small sample size (nine affected and five carrier sheep) and the relatively sparse genome coverage.

The recessive inheritance pattern of the affected Merino sheep suggested the causative variant would be homozygous in affected sheep. Whole genome sequencing was performed on two affected sheep to a depth of 50× each. The WGS data for the affected sheep were then compared to the Texel sheep reference genome [11]. This identified about 4 million homozygous variants common to both affected sheep and not in the Texel reference genome.

Filtering for variants in exons or introns of known protein coding genes reduced this list to ~ 1.2 million variants, including a high number of potentially damaging variants. These included 660 stop-gain mutations in protein-coding genes. It was suspected most of these variants represented differences between the Texel and Merino sheep genomes. We therefore used low coverage (5×) WGS data from an additional four affected and eight unaffected, unrelated control Merino sheep to filter the remaining potential disease-causing variants. As poor sequencing coverage limited accuracy and zygosity calling, these data were used only to support or refute the potential pathogenicity of high-quality homozygous variants identified in the two 50× WGS datasets.

We next reasoned that none of the control Merino sheep would have the pathogenic variant, since carrier frequencies are likely to be very low or non-existent among contemporary Merino sheep. OCPMD has not been recorded outside our research flock since the 1970s [2]. We also reasoned that the pathogenic variant would be homozygous in the additional affected sheep and thus no reads in the affected sheep would contain the reference allele at the pathogenic locus. Finally, we required that the variant lie in a region with read coverage ≥ 1× in at least six of the controls and three additional affected sheep (≥ 75% of each subset).

Variants in the resulting set were reviewed and deletion of the canonical “G” in the splice donor site (+ 1) of intron 13 of the *TNNT1* gene was identified as possibly causal. *TNNT1* was an attractive candidate for further validation due to association with nemaline myopathy in humans [22–28] and its type I myofibre specificity. Sanger sequencing of two wild-type, nine carrier, and seven affected sheep confirmed that the mutation segregated completely with the disease phenotype (Additional file 4: Fig. S3). Based on the published sheep *TNNT1* cDNA sequence [18], the variant annotation is KT218690 c.614 + 1delG.

### RT-PCR demonstrated mRNA intron 13 inclusion in both carrier and affected sheep

Mutations at splice donor or acceptor sites can lead to exon skipping, intron retention, or insertions and deletions due to utilization of cryptic splice sites [29]. We therefore suspected one of these mechanisms would disrupt the protein sequence and function of TNNT1 in sheep with the c.614 + 1delG variant. RT-PCR was used to identify potential splicing defects in type I myofibre-predominant muscles (vastus intermedius) from affected sheep.

**Fig. 2:**
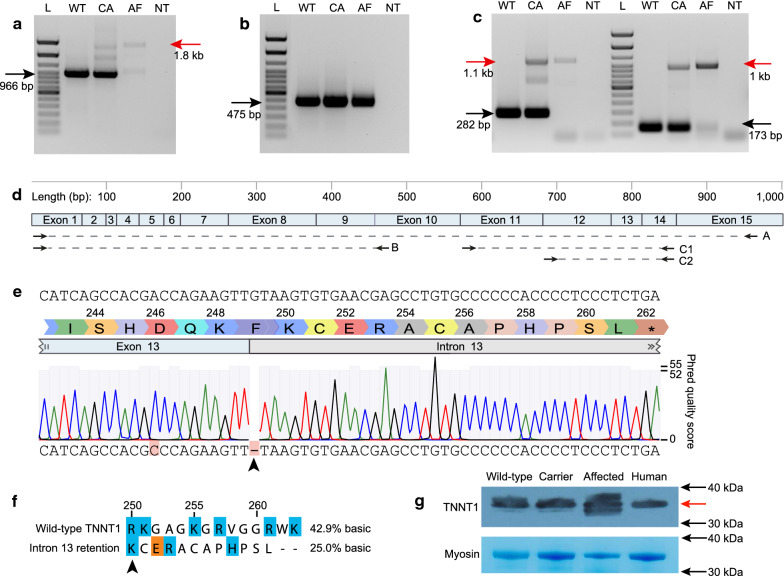
The *TNNT1* c.614 + 1delG mutation causes intron 13 inclusion but does not reduce protein abundance. Agarose gels of RT-PCR products derived from type I myofibre-predominant vastus intermedius muscles of wild-type (WT), carrier (CA) and affected (AF) sheep for: **a** full-length TNNT1 (exon 1–15), **b** region encoding the protein N-terminus (exons 1–10), and **c** region encoding the C-terminus (exons 11–14). Expected (normal) product sizes are indicated by black arrows, and unexpected products by red arrows. NT = no template control. Ladder (L) = 100 bp TrackIT (Thermo Fisher, USA). **d** Schematic of primer sites and amplicons relative to the *Ovis aries* v3.1 *TNNT1* genomic sequence. Amplicons are labelled relative to which panel they appear in. The full-length cDNA is 1001 bp and contains 15 exons. Translation starts in exon 2 and terminates in exon 14. **e** Sanger sequencing chromatogram of RT-PCR product from affected sheep, aligned to the published ovine TNNT1 cDNA sequence (Accession # KT218690, top sequence). Mismatches are highlighted in red. The causative single nucleotide deletion is indicated by an arrow. Base call quality (Phred quality score) is indicated by blue/grey bars at each position. Only partial intron 13 sequence is shown, up until the new stop codon in intron 13. The total size of intron 13 is 809 bp. Newly-encoded amino acids are indicated above the exon/intron annotations using the single letter amino acid code. Numbers represent amino acid position. **f** Pairwise comparison of the terminal 14 amino acids from wild-type TNNT1 and the amino acid sequence produced from intron 13 retention. Basic (blue) and acidic (orange) residues are highlighted. Basic residues within this region are known to be important for activation of TNNT1 by Ca^2+^ [31]. **g** Western blot of TNNT1 (HPA058448) from vastus intermedius muscle samples from 1-year old wild-type, carrier, and affected sheep. Human control is a skeletal muscle sample from quadriceps of a healthy individual. Red arrow indicates expected protein size for all samples (~ 33 kDa). Coomassie-stained myosin gel band (total myosin) was used as a loading control

RT-PCR using primers that should amplify the full-length *TNNT1* cDNA generated a product of the expected size (966 bp) from wild-type and carrier sheep, but not from muscle cDNA from affected sheep (Fig. 2a). Rather, the predominant product amplified from affected samples was ~ 1.8 kb in size (Fig. 2a). This size disparity was consistent with retention of intron 13 (+ 810 bp; based on the Oar v3.1 assembly). This product was also observed in carrier samples, but never in wild-type samples (Fig. 2a).

To further confirm the *TNNT1* c.614 + 1delG deletion results in intron 13 retention, we performed RT-PCR using primers that target the N- and C-terminal-encoding regions. N-terminal products were all the expected size and comparable intensity across wild-type, carrier and affected samples (Fig. 2b). However, no wild-type RT-PCR products could be detected in affected samples when targeting exons flanking intron 13 (Fig. 2c). Similar to full-length RT-PCR results, an ~ 800 bp larger product was observed in all affected samples (Fig. 2c) and correlated precisely with the size of intron 13 (+ 810 bp).

### Intron 13 retention disrupts the highly conserved 14 amino acid C-terminus of TNNT1

As RT-PCR results were consistent with inclusion of intron 13, we TA-cloned each PCR sample and sequenced multiple clones. As expected, enlarged (+ 800 bp) products from carrier and affected samples contained the c.614 + 1delG donor splice site deletion and the entire intron 13 (Fig. 2e). The retention of this intron would result in translation of 12 amino acids followed by a stop codon (Fig. 2e), such that the terminal 14 amino acids encoded by exon 14 would be replaced with a new series of 12 amino acids (Fig. 2f).

Importantly, the last 14 amino acids of TNNT1 (encoded by exon 14) are highly conserved across mammalian species (Additional file 5: Fig. S4) and also in fast and cardiac troponin T isoforms [30]. Although the mutant transcripts are not predicted to substantially alter the size of the resulting protein (wild-type: 263 amino acids, 31.4 kDa; intron 13 retention: 261 amino acids, 31.2 kDa), the amino acid composition and properties of the C-terminus are affected (Fig. 2f). Notably, the number of basic residues within this conserved 14 amino acid functional domain is substantially reduced (6/14 in wild-type, 3/14 with intron retention) (Fig. 2f). The importance of basic residues within this domain has been previously reported for cardiac troponin T [31].

### TNNT1 protein abundance is not reduced in affected sheep

Although we did not expect to see any significant differences in the size of the mutant protein compared to wild-type based on calculated coding sequence lengths, we wished to determine whether abnormal splicing affects protein abundance (e.g. due to decreased protein stability or degradation, as reported in human cases [32]). Western blot analysis showed no reduction in TNNT1 protein size or abundance compared to both wild-type and carrier sheep in vastus intermedius or other affected muscles (Fig. 2g, Additional file 6: Fig. S5). In addition to the major isoform (~ 33 kDa; as represented in the human control skeletal muscle), smaller and larger isoforms were also observed in samples from affected sheep (Fig. 2g, Additional file 6: S5A). Altogether, these results indicated that reduced (or absent) TNNT1 protein is not the cause of OCPMD.

### No overt differences in TNNT1 localization were observed in type I (slow) myofibres from affected sheep

**Fig. 3:**
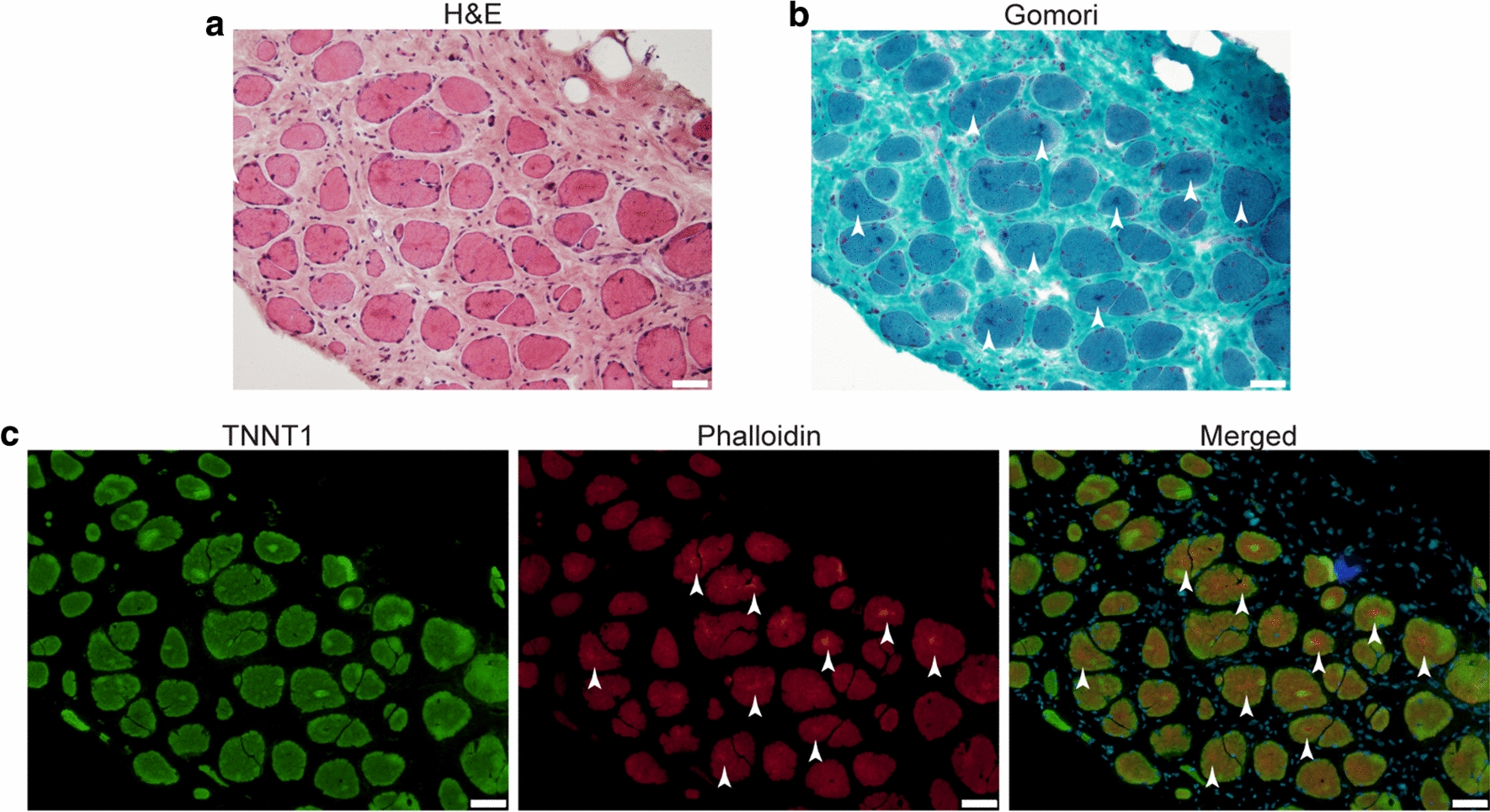
TNNT1 does not co-localize with actin-positive aggregates in affected muscles. Immunohistochemical analysis of soleus muscle from an 8-year old affected sheep. **a** Hematoxylin and eosin (H&E) staining. **b** Gomori trichrome staining. Multiple sarcoplasmic protein aggregates are visible (arrows). **c** Staining for TNNT1 (green), F-actin (phalloidin, red) and nuclei (blue). Actin-positive aggregates are marked with arrows. TNNT1 does not co-localize with these aggregates (see Merged). Scale bars = 50 µm

Given we observed no significant reduction in TNNT1 protein abundance, and that C-termini are often important for correct localization of proteins [33], we next hypothesized that the disruption of the terminal 14 amino acids of TNNT1 may influence its localization and hence, its function. However, we observed no overt abnormalities in TNNT1 localization in affected (type I myofibre-predominant) skeletal muscles (Fig. 3). We also co-stained for F-actin using phalloidin but did not find any evidence of TNNT1 sequestration within F-actin positive aggregates (Fig. 3c).

### Ca^2+^-sensitivity is increased in skinned myofibres from affected sheep

Crystal structures of Ca^2+^-bound cardiac troponin T previously indicated that the C-terminal region forms an unstructured domain that interacts with tropomyosin, and that this interaction is modulated by calcium concentration [34]. Since the c.614 + 1delG variant would completely alter this domain and thus perhaps disrupt the normal interaction with tropomyosin, we suspected that OCPMD may be caused by impaired regulation of skeletal muscle contraction.

**Fig. 4:**
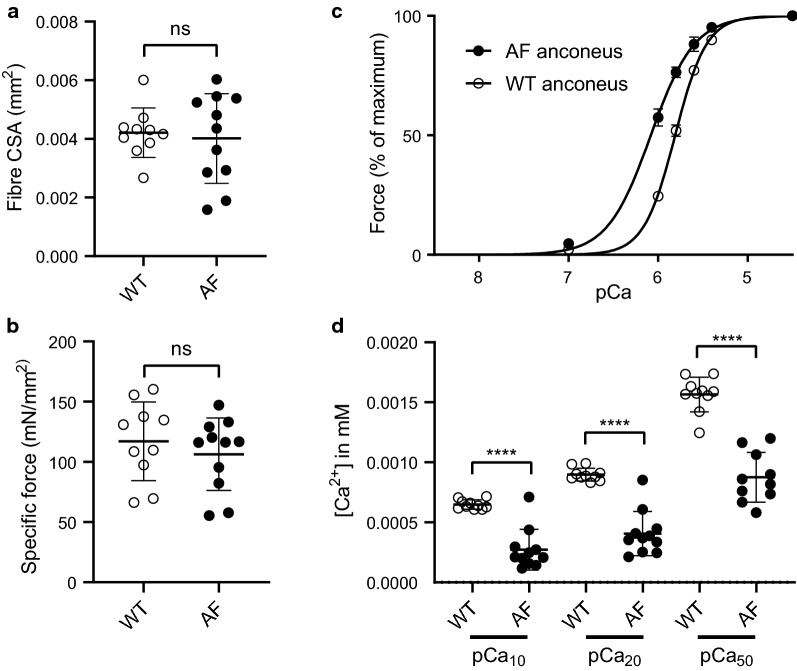
Type I OCPMD myofibres initiate force generation at twofold lower Ca^2+^ concentration than wild-type fibres. Physical and physiological properties of single type I fibres from type I myofibre-predominant muscles (anconeus) of wild-type (WT) and affected (AF) sheep, including (**a**) fibre cross-sectional area (CSA), **b** maximal specific force, **c** force-pCa curve relating force generation to − log(10) of the calcium concentration (pCa), and (**d**) pCa plot of calcium concentration (mM) required to generate 10% (pCa_10_), 20% (pCa_20_), and 50% (pCa_50_) of maximal force. Statistical differences between wild-type and affected samples were assessed using a two-tailed, unpaired t-test; ns: not significant, ****p < 0.0001

Analysis of individual type I-myofibres from affected sheep indicated no significant decrease in cross-sectional area or specific force of myofibres compared to controls (Fig. 4a,b). However, OCPMD myofibres displayed a significant and substantial leftward shift of the pCa-force relationship. Specifically, the pCa_50_ (the Ca^2+^ concentration required for 50% of maximal force) was significantly reduced for affected myofibres (Fig. 4c). This represents an approximately twofold decrease in the Ca^2+^ concentration required to achieve 50% maximal force (wild-type: 0.00157 ± 0.00014 mM, affected: 0.00088 ± 0.00021 mM, *p* < 0.0001) (Fig. 4d). The same was also true at 10% and 20% of maximal force (*p* < 0.0001 for both) (Fig. 4d). Given that muscle function is typically between 10 and 65% of its maximal force during normal activity [35], this represents a physiologically relevant increase in Ca^2+^-sensitivity of type I myofibres in OCPMD sheep.

## Discussion

OCPMD was initially described in the 1960s and 1970s [1, 2]. The Western Australian flock studied here was characterized by Richards and Passmore and their colleagues in a series of papers in the 1980s [3–7]. Histopathology shows that type I myofibre-prominent muscles are specifically affected in OCPMD. Myofibres are replaced with fat, consistent with a dystrophic process, but the affected myofibres also contain central sarcomeric protein aggregates consistent with human congenital myopathies such as nemaline myopathy [4–6]. The recessive inheritance of the disease demonstrated it was a genetic disorder. However, because of the different pathological features, it was never clear whether OCPMD represents a model of muscular dystrophy or a myopathy, since some myopathies may exhibit dystrophic features [36, 37].

The molecular cause of OCPMD has not been elucidated until now. Here, we identify the genetic cause of OCPMD in the Western Australian flock [2] as a single nucleotide deletion within the canonical splice donor site (+ 1) in intron 13 of the *TNNT1* gene (KT218690 c.614 + 1delG). This suggests that OCPMD is a nemaline myopathy with dystrophic features, since variants in *TNNT1* have been previously associated with recessive [22–26, 28, 32, 38, 39] and dominant [27] nemaline myopathy across multiple human populations. However, as the histopathology also shows features of other myopathies (e.g. protein aggregation, dystrophic changes), we believe it is most appropriate to class OCPMD as a model of ‘TNNT1 congenital myopathy’ rather than nemaline myopathy, specifically.

Location of the causative variant in *TNNT1* fits with the predominance of pathology in type I myofibres of OCPMD sheep. *TNNT1* encodes the slow skeletal muscle troponin T (ssTnT) which is a vital component of the troponin complex that is integral to Ca^2+^-regulation of skeletal muscle contraction [40]. Specifically, the troponin complex confers Ca^2+^-sensitivity to striated muscle actomyosin ATPase activity [30, 41]. In mice, ssTnT deficiency causes atrophy of type I myofibres and decreased tolerance to fatigue [42]; both of which are key features of the OCPMD model. Further targeted gene studies in mice also show knockdown of ssTnT causes myopathic phenotypes, including significantly decreased type I myofibre size and quantity in type I myofibre-predominant muscles [43]. Altogether, *TNNT1* as the causative gene for OCPMD is concordant with phenotypes previously described in the literature.

Although the OCPMD c.614 + 1delG variant disrupts the TNNT1 C-terminus (Fig. 2e,f), we did not observe any reduction in TNNT1 protein levels (Fig. 2g) or altered localization in myofibres from affected sheep (Fig. 3). The presence of smaller protein bands in affected samples by western blot analysis may indicate degradation products [44]. However, since multiple TNNT1 isoforms have been previously reported in sheep [45] and other organisms [46] we suspect this is more likely to indicate a compensatory mechanism whereby alternative transcript isoforms are upregulated in affected muscles, as has been reported in cases of human *TNNT1* nemaline myopathy [27].

Given that we did not observe any overt changes in TNNT1 abundance or localization by western blot or immunohistochemistry, we suspected that the pathology was caused by disruption of the function of the exon 14-encoded C-terminus. This 14 amino acid region is highly conserved, not only between mammalian species (Additional file 5: Fig. S4), but also between the human cardiac (TNNT2) and fast skeletal muscle (TNNT3) isoforms of troponin T (TnT) [30]. Although this region is difficult to crystallize (and therefore is absent from most crystal structures) [34], the effects of deleting this region (Δ14 TnT) have been previously examined in the context of cardiac troponin T [47, 48]. Gafurov et al. showed that the Δ14 TnT deletion increases Ca^2+^ sensitivity without significantly affecting skeletal muscle force [48]. Franklin et al. further showed that Δ14 TnT protein has elevated actin-activated ATPase activity in the presence and absence of Ca^2+^ [47]. More recently, Johnson et al. showed that basic residues within this region in cardiac troponin T are critical for the regulation of cardiac muscle contraction [31]. Although these studies examined cardiac troponin T, the high conservation of the C-terminus between troponin isoforms strongly suggests that the mechanism is also likely to be applicable to skeletal muscle troponin T. The OCPMD mutation significantly alters the number and position of basic residues within the C-terminal region (Fig. 2F). Therefore, it is likely that scrambling and partial truncation of the C-terminus in OCPMD sheep impacts TNNT1 Ca^2+^ sensitivity.

To assess whether impaired Ca^2+^ sensitivity could be the causative disease mechanism in OCPMD sheep, we examined the physiological properties of type I myofibre bundles and/or single myofibres from affected sheep. In line with findings from cardiac TnT [47, 48], C-terminal disruption did not impact force, but did increase Ca^2+^ sensitivity approximately twofold (Fig. 4). As TnT is not known to directly bind Ca^2+^ it is most likely that the effect is exerted via interaction between the TnT C-terminus and the troponin Ca^2+^-binding subunit, troponin C [49, 50]. Although saturating Ca^2+^ normally gives about 30% of the maximum possible ATPase activity of sarcomeres [49], this disruption could feasibly double this activation. As these experiments were all performed within the normal activation range of muscle (10–60%) and at physiological Ca^2+^ concentrations, these data can be extrapolated to in vivo muscle function and therefore define a pathogenic mechanism for the causative *TNNT1* variant. That is, the frequency and timing of muscle contraction becomes partially decoupled from intracellular Ca^2+^ transients.

Various myopathies are known to be caused by this decoupling mechanism and result from variants in other skeletal muscle genes involved in the Ca^2+^-dependent contraction response, including *TPM2* [51, 52], *TPM3* [53], and *ACTA1* [54, 55]. These mutations produce hypercontractile and/or hypertonic phenotypes by disruption of the actin-tropomyosin interface. These features are consistent with the observed characteristics of OCPMD sheep, including abnormal gait and skeletal muscle stiffness. As the OCPMD variant affects TNNT1 but not the cardiac or fast skeletal muscle isoforms, this also explains why only type I (slow) myofibres/muscles are affected. Altogether, we propose that disruption of the TNNT1 C-terminal results in type I myofibre-predominant muscles that are unable to properly regulate muscle relaxation and contraction (via actin) in response to Ca^2+^ levels.

In comparison to human variants, the only confirmed human TNNT1 nemaline myopathy cases that show production of intact TNNT1 protein are autosomal dominant cases caused by a missense variant within one of the two highly conserved tropomyosin binding sites [27]. Rather, the majority of human cases of *TNNT1* nemaline myopathy are caused by autosomal recessive nonsense mutations [22, 24–26, 28, 32, 38] that prevent incorporation of TNNT1 into the myofilament, leading to rapid protein degradation [32]. This results in atrophy of type I myofibres and causes a range of clinical features including a specific tremor in cases of Amish nemaline myopathy [22]. Such cases are typically quite severe and lead to death before 2 years of age.

However, a relevant human case of *TNNT1* nemaline myopathy describes a compound heterozygous splice site change (resulting in exon 8 deletion) and an exon 14 deletion in a Dutch pedigree [23]. Using engineered TNNT1 mutants, Amarasinghe et al. subsequently showed that the exon 8 deletion, and various known nonsense mutations, act by diminishing binding of the troponin complex to tropomyosin [56]. Although not examined in the above studies, we suspect that the exon 14 deletion would result in intact TNNT1 protein with altered function as it involves the same 14 amino acids impacted by the OCPMD variant. This may partially explain the slightly milder phenotype of these patients compared to more common truncating variants. Altogether, it is apparent that large truncating TNNT1 variants that affect tropomyosin binding sites lead to a more severe phenotype than variants which allow production of intact TNNT1. This difference may also explain the lack of dystrophic features in cases of human *TNNT1* nemaline myopathy compared to the OCPMD model.

## Conclusion

Here, we identify the genetic cause of the well-characterized model of ovine congenital progressive muscular dystrophy from Western Australia and re-classify it as a congenital myopathy with dystrophic features. This model is useful as it mimics many pathological features of human skeletal muscle disease and exhibits features not frequently seen together in human patients. Type I myofibre-predominant skeletal muscles are specifically affected, allowing studies on differential muscle involvement. Our identification of the causative mutation in *TNNT1* makes the OCPMD sheep an attractive large animal model for better understanding the pathobiology of human TNNT1 myopathy. Moreover, this model could prove valuable for testing of future therapies (e.g. viral treatment), especially considering the body size and skeletal muscle mass of sheep more closely matches humans than other animal models [57].

## Supplementary information

**Additional file 1: Figure S1.**Complete pedigree of sheep used in the current study.

**Additional file 2: Table S1.** RT-PCR and sequencing primers used for *TNNT1* transcript analyses.

**Additional file 3: Figure S2.** Predominantly type I (slow) myofibres show pathology in affected sheep. Histology of vastus intermedius muscles from wild-type, carrier, and affected sheep at 1 year of age (1 y.o.) and 8 years of age (8 y.o.). **a** Hematoxylin and eosin (H&E) staining; shows marked variation in myofibre diameter, shape and increased spacing between myofibres in affected sheep. **b** Gomori trichrome staining; white areas are indicative of replacement of myofibres by adipocytes. Scale bars = 50 µm.

**Additional file 4: Figure S3.** Sanger sequencing of c.614+1delG in wild-type, carrier and OCPMD-affected sheep. Sanger sequencing across the c.614+1delG site (black arrow/blue line). Deletion is absent in wild-type sheep, heterozygous in carriers (causing a frameshift), and homozygous in affected sheep.

**Additional file 5: Figure S4.** The terminal 14 amino acids of TNNT1 are absolutely conserved across multiple mammalian species. Clustal Omega alignment of TNNT1 amino acid sequences across 6 different mammalian species. The exons that encode each region of the protein are labelled. Of note, exon 14 encodes the terminal 14 amino acids of TNNT1, which produce an intrinsically disordered domain that binds to tropomyosin [34]. Protein sequences used for alignments were as follows: sheep (*Ovis aries*): AMR55385 (published AA sequence from K218690 CDS), human (*Homo sapiens*): NP_0011196044 (NCBI RefSeq), cow (*Bos taurus*): NP_776899 (NCBI RefSeq), mouse (*Mus musculus*): NP_001264833 (NCBI RefSeq), rat (*Rattus norvegicus*): NP_001264191 (NCBI RefSeq), and dog (*Canis lupus familiaris*): XP_005616225 (NCBI predicted).

**Additional file 6: Figure S5.** Replicate western blots show no significant increase in TNNT1 protein in affected sheep. A) Replicate western blots for TNNT1 (HPA058448) from vastus intermedius of 1-year old wild-type (WT), carrier (CA), and affected (AF) sheep. Human control is a skeletal muscle sample from quadriceps of a healthy individual. Coomassie-stained myosin gel band (total myosin) was used as a loading control. Top and bottom of each image represent 40 and 30 kDa bounds, respectively. Densitometry was performed using ImageJ and resulting values are indicated below each lane; TNNT1 signal was normalized to total myosin and is presented relative to the matched wild-type. **B)** Relative TNNT1 protein abundance, as calculated from blots in **(A)**. Statistical differences were assessed using a two-tailed, unpaired t-test. ns = not significant. **C)** Western blot from anconeus and triceps of age-matched wild-type and affected sheep. Densitometry was performed as above.

## Data Availability

The majority of the data generated or analysed during this study are included in this published article and its supplementary information files. Any additional datasets are available from the corresponding author on reasonable request.

## Abbreviations

ACTA1: Actin Alpha 1, skeletal muscle
ATP: Adenosine triphosphate
BSA: Bovine serum albumin
cDNA: Complementary DNA
CDS: Coding sequence
H&E: Haematoxylin and eosin
kDa: Kilodalton
OCPMD: Ovine congenital progressive muscular dystrophy
PBS: Phosphate buffered saline
PCR: Polymerase chain reaction
RT-PCR: Reverse transcriptase polymerase chain reaction
SDS: Sodium dodecyl sulphate
SNP: Single nucleotide polymorphism
ssTnT: Slow skeletal muscle troponin T
TNNT1: Troponin T1, slow skeletal type
TNNT2: Troponin T2, cardiac type
TNNT3: Troponin T3, fast skeletal type
TnT: Troponin T
TPM2: Tropomyosin 2
TPM3: Tropomyosin 3
WA: Western Australia
WGS: Whole genome sequencing
