# Supplementary figures and images for: Ovine congenital progressive muscular dystrophy (OCPMD) is a model of TNNT1 congenital myopathy
Source: Acta Neuropathol Commun. 2020 Aug 20;8:142. doi: 10.1186/s40478-020-01017-1 (PMC7441672; doi:10.1186/s40478-020-01017-1)

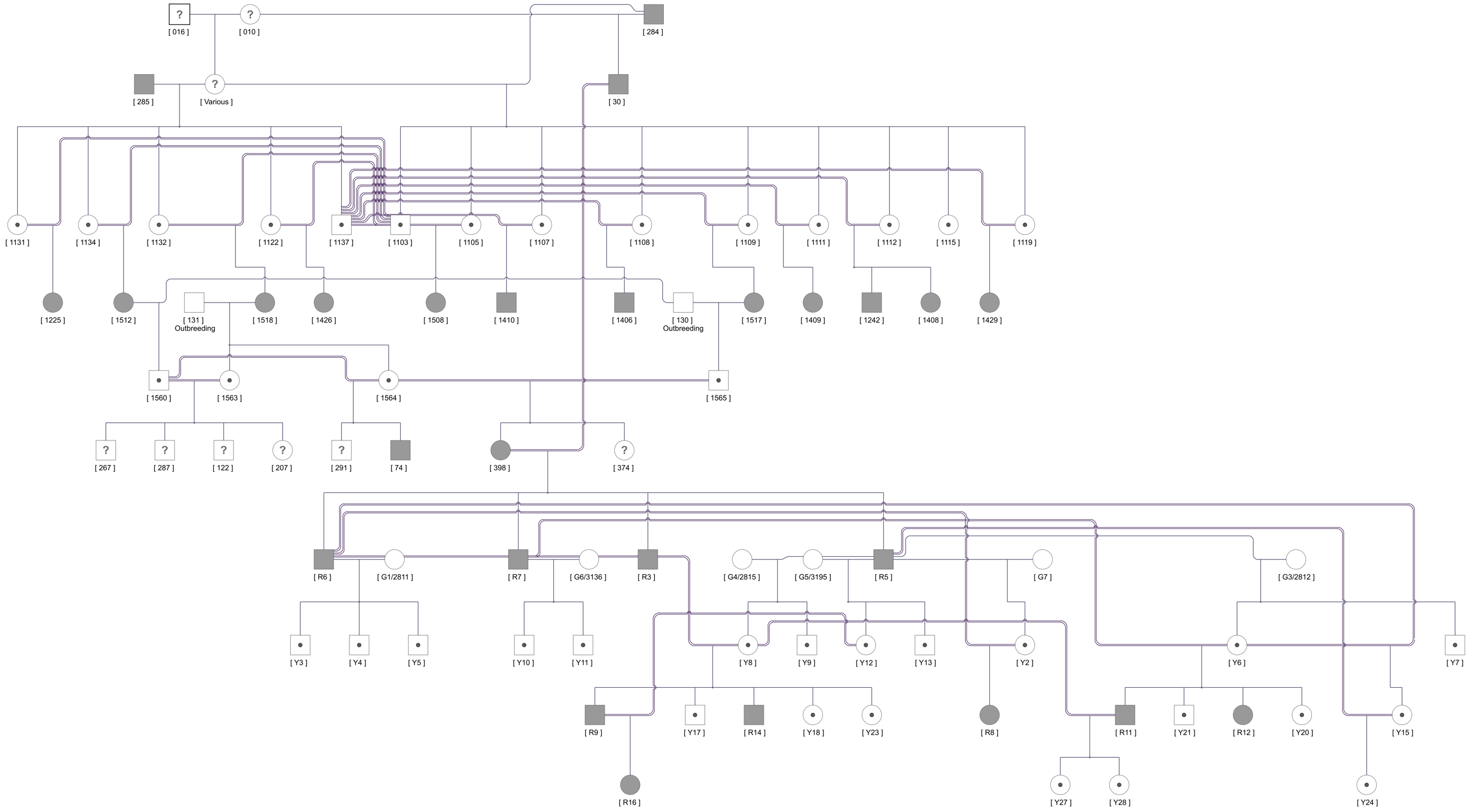

Supplement: Supplementary file 1 — Additional file 1: Figure S1.Complete pedigree of sheep used in the current study. [file 40478_2020_1017_MOESM1_ESM.pdf]

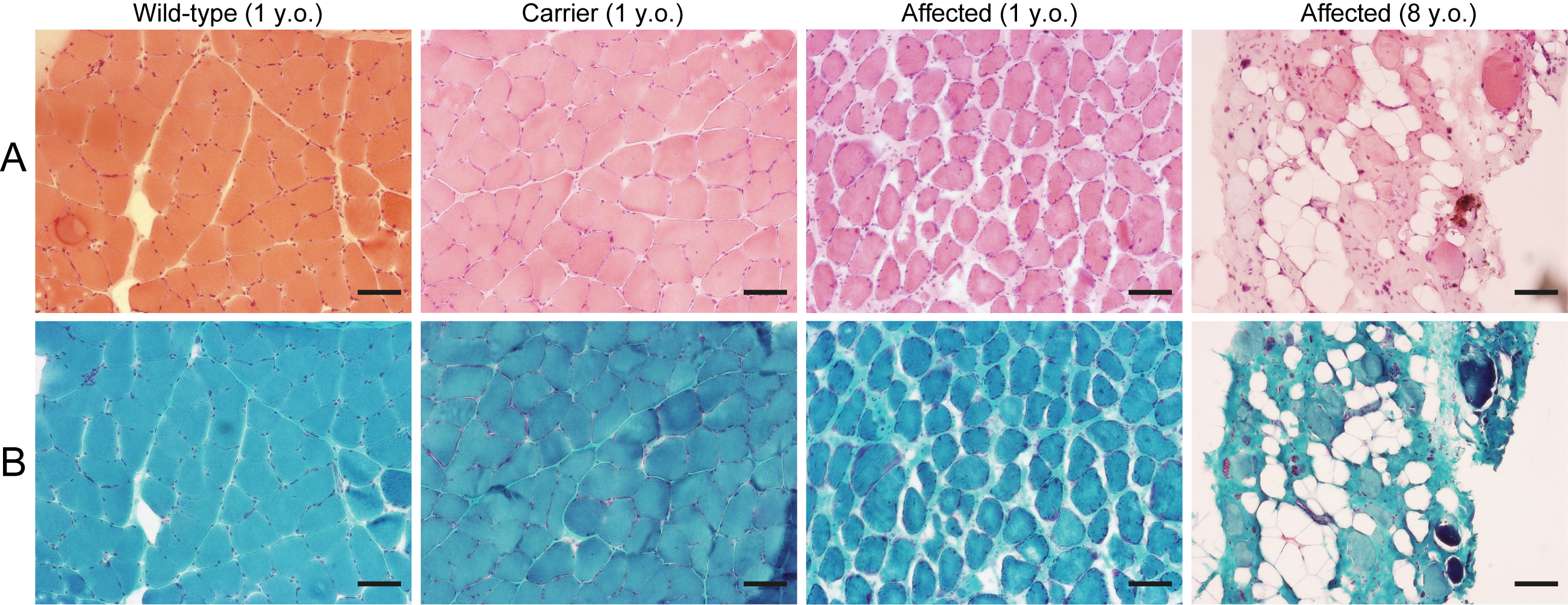

Supplement: Supplementary file 3 — Additional file 3: Figure S2. Predominantly type I (slow) myofibres show pathology in affected sheep. Histology of vastus intermedius muscles from wild-type, carrier, and affected sheep at 1 year of age (1 y.o.) and 8 years of age (8 y.o.). a Hematoxylin and eosin (H&E) staining; shows marked variation in myofibre diameter, shape and increased spacing between myofibres in affected sheep. b Gomori trichrome staining; white areas are indicative of replacement of myofibres by adipocytes. Scale bars = 50 µm. [file 40478_2020_1017_MOESM3_ESM.tif]

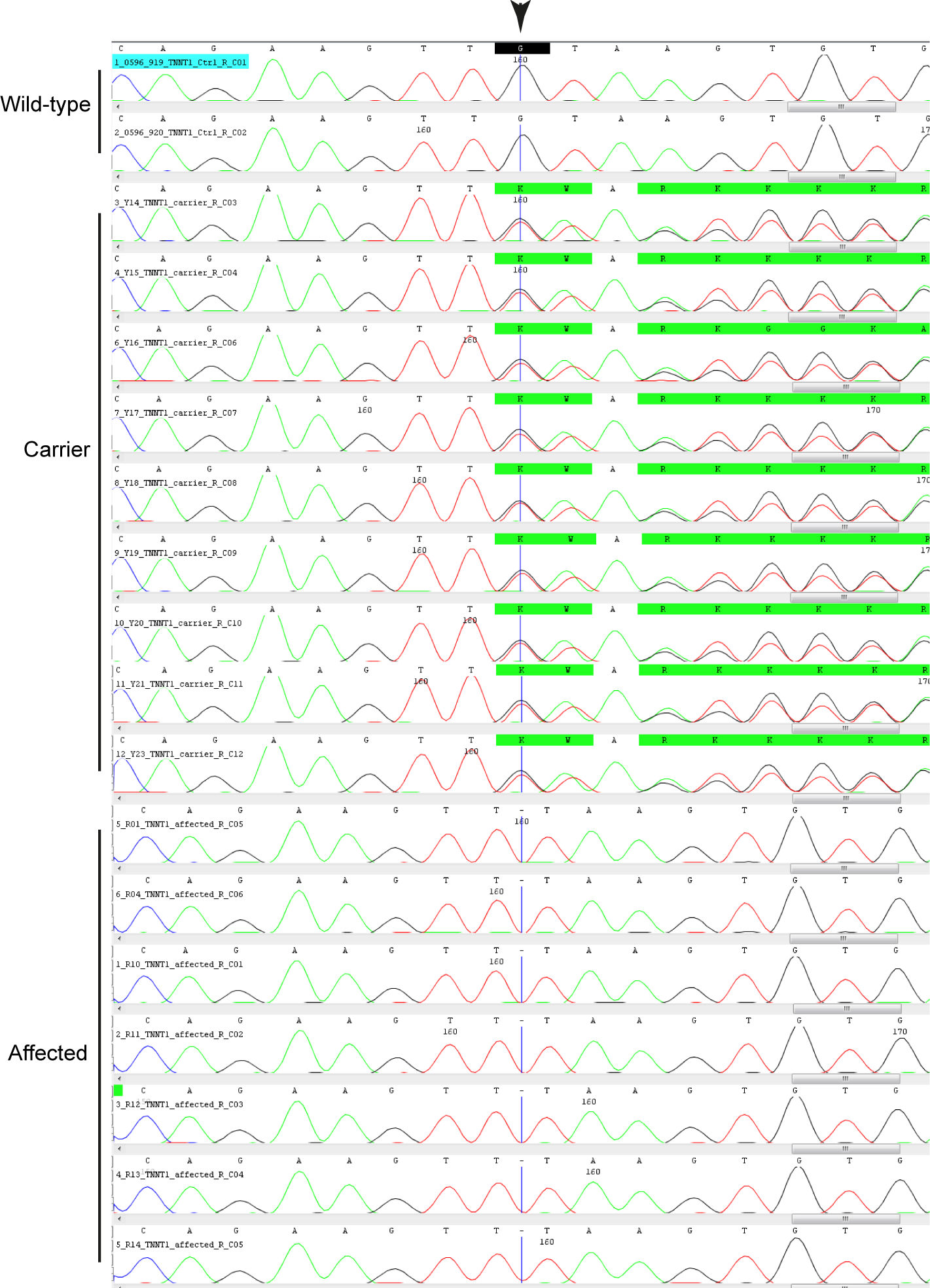

Supplement: Supplementary file 4 — Additional file 4: Figure S3. Sanger sequencing of c.614+1delG in wild-type, carrier and OCPMD-affected sheep. Sanger sequencing across the c.614+1delG site (black arrow/blue line). Deletion is absent in wild-type sheep, heterozygous in carriers (causing a frameshift), and homozygous in affected sheep. [file 40478_2020_1017_MOESM4_ESM.tif]

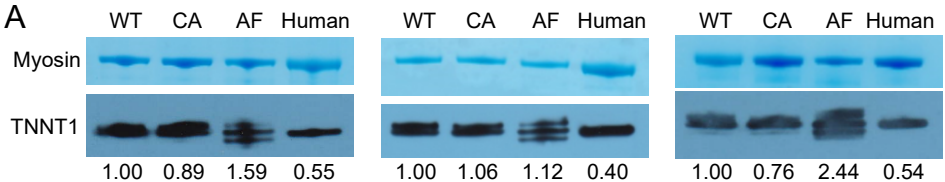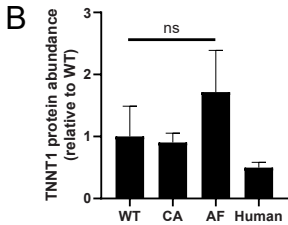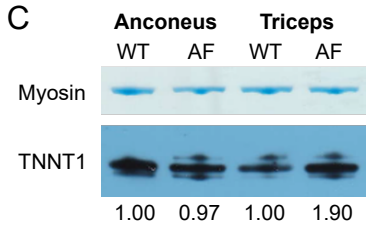

Supplement: Supplementary file 6 — Additional file 6: Figure S5. Replicate western blots show no significant increase in TNNT1 protein in affected sheep. A) Replicate western blots for TNNT1 (HPA058448) from vastus intermedius of 1-year old wild-type (WT), carrier (CA), and affected (AF) sheep. Human control is a skeletal muscle sample from quadriceps of a healthy individual. Coomassie-stained myosin gel band (total myosin) was used as a loading control. Top and bottom of each image represent 40 and 30 kDa bounds, respectively. Densitometry was performed using ImageJ and resulting values are indicated below each lane; TNNT1 signal was normalized to total myosin and is presented relative to the matched wild-type. B) Relative TNNT1 protein abundance, as calculated from blots in (A). Statistical differences were assessed using a two-tailed, unpaired t-test. ns = not significant. C) Western blot from anconeus and triceps of age-matched wild-type and affected sheep. Densitometry was performed as above. [file 40478_2020_1017_MOESM6_ESM.pdf]
