# Supplementary material for: Ovine congenital progressive muscular dystrophy (OCPMD) is a model of TNNT1 congenital myopathy
Source: Acta Neuropathol Commun. 2020 Aug 20;8:142. doi: 10.1186/s40478-020-01017-1 (PMC7441672; doi:10.1186/s40478-020-01017-1)
Supplement: Supplementary file 2 — Additional file 2: Table S1. RT-PCR and sequencing primers used for TNNT1 transcript analyses. [file 40478_2020_1017_MOESM2_ESM.docx]

**Table S1. RT-PCR and sequencing primers used for *TNNT1* transcript analyses.**

| Primer | Sequence (5' − 3') | Tm (°C)^a^ | Position^b^ | Amplicon |
| --- | --- | --- | --- | --- |
| TNNT1 cDNA F | GATTTCTCCCCACCACTCT | 52.5 | +/ 21 | 966 bp |
| TNNT1 cDNA R | CATTTATTTCAAGCCCCAG | 48.3 | –/ 950 |  |
| TNNT1 N-ter F | GATTTCTCCCCACCACTCT | 52.5 | +/ 21 | 475 bp |
| TNNT1 N-ter R | TTCCAGCGGCCTCCGA | 55.3 | –/ 458 |  |
| TNNT1 C-ter 1 F | AGAACAGAAGCGAGGGAAGC | 56.5 | +/ 593 | 282 bp |
| TNNT1 C-ter 1 R | TTCCAGCGGCCTCCGA | 57.4 | –/ 840 |  |
| TNNT1 C-ter 2 F | GAGAAGGCCCAGGAACTGTC | 56.4 | +/ 702 | 173 bp |
| TNNT1 C-ter 2 R | TTCCAGCGGCCTCCGA | 57.4 | –/ 840 |  |
| TNNT1 C-ter seq F | GAAAGTGCGTATCCTGTCTGAG | 55.1 | +/ 634 | Sequencing |
| M13 Forward | GTAAAACGACGGCCAG | 49.6 | - | Sequencing |
| M13 Reverse | CAGGAAACAGCTATGAC | 46.1 | - | Sequencing |

^a^Primer melting temperatures (Tm) were calculated using Primer3.

^b^Primer positions are relative to the published ovine TNNT1 cDNA sequence (KT218690).
