# Supplementary material for: Ovine congenital progressive muscular dystrophy (OCPMD) is a model of TNNT1 congenital myopathy
Source: Acta Neuropathol Commun. 2020 Aug 20;8:142. doi: 10.1186/s40478-020-01017-1 (PMC7441672; doi:10.1186/s40478-020-01017-1)
Supplement: Supplementary file 5 — Additional file 5: Figure S4. The terminal 14 amino acids of TNNT1 are absolutely conserved across multiple mammalian species. Clustal Omega alignment of TNNT1 amino acid sequences across 6 different mammalian species. The exons that encode each region of the protein are labelled. Of note, exon 14 encodes the terminal 14 amino acids of TNNT1, which produce an intrinsically disordered domain that binds to tropomyosin [34]. Protein sequences used for alignments were as follows: sheep (Ovis aries): AMR55385 (published AA sequence from K218690 CDS), human (Homo sapiens): NP_0011196044 (NCBI RefSeq), cow (Bos taurus): NP_776899 (NCBI RefSeq), mouse (Mus musculus): NP_001264833 (NCBI RefSeq), rat (Rattus norvegicus): NP_001264191 (NCBI RefSeq), and dog (Canis lupus familiaris): XP_005616225 (NCBI predicted). [file 40478_2020_1017_MOESM5_ESM.pdf]

```
TNNT1_dog      MSDTEEQEYEEEQPEEEEAAEEEEEEAAEEPEPVAEREEERPKP-RPVVPPLIPPKIPEGE   59
TNNT1_sheep    MSDAEEQEYEEEQPEEEEAAEEEEEEAPEEPEPAAEREEERPKPSRPVVPPLIPPKIPEGE   60
TNNT1_human    MSDTEEQEYEEEQPEEE-AAEEEEEEAPEEPEPVAEPEEERPKPSRPVVPPLIPPKIPEGE   59
Tnnt1_rat      MSDTEEQEYEEEQAEDEEAV--------------EEEEEERPKPSRPVVPPLIPPKIPEGE   47
TNNT1_cow      MSDAEEQEYEEEQPEEEEAAEEEEEEAPEEPEPAAEPEEERPKPSRPVVPPLIPPKIPEGE   60
Tnnt1_mouse    MSDTEEQEYEEEQAEDEEAV--------------EEEEEERPKPSRPVVPPLIPPKIPEGE   47
               ***:********* *:* *.               * ******* ****************
                  Exon 2     Exon 3  Exon 4         Exon 5    Exon 6      Exon 7

TNNT1_dog      RVDFDDIHRKRMEKDLLELQTLIDVHFEQRKKEEEELIALKERIERRRAERAEQQRFRTE   119
TNNT1_sheep    RVDFDDIHRERMEKDLLELQTLIDVHFEQRKKEEEELVALKERIERRRAERAEQQRFRTE   120
TNNT1_human    RVDFDDIHRKRMEKDLLELQTLIDVHFEQRKKEEEELVALKERIERRRSERAEQQRFRTE   119
Tnnt1_rat      RVDFDDIHRKRMEKDLLELQTLIDVHFEQRKKEEEELIALKDRIERRRAERAEQQRFRTE   107
TNNT1_cow      RVDFDDIHRKRMEKDLLELQTLIDVHFEQRKKEEEELVALKERIERRRAERAEQQRFRTE   120
Tnnt1_mouse    RVDFDDIHRKRMEKDLLELQTLIDVHFEQRKKEEEELIALKDRIERRRAERAEQQRFRTE   107
               *********:***************************:***:******:***********
                                       Exon 8                      Exon 9

TNNT1_dog      KERERQAKLAEEKMRKEEEEAKKRAEDDAKKKKVLSNMGAHFGGYLVKAEQKRGKRQTGR   179
TNNT1_sheep    KERERQAKLAEEKMRKDEEEAKKRAEDDAKKKKVLSNMGAHFGGYLVKAEQKRGKRQTGR   180
TNNT1_human    KERERQAKLAEEKMRKEEEEAKKRAEDDAKKKKVLSNMGAHFGGYLVKAEQKRGKRQTGR   179
Tnnt1_rat      KERERQAKLAEEKMRKEEEEAKKRAEDDAKKKKVLSNMGAHFGGYLVKAEQKRGKRQTGR   167
TNNT1_cow      KERERQAKLAEEKMRKEEEEAKKRAEDDAKKKKVLSNMGAHFGGYLVKAEQKRGKRQTGR   180
Tnnt1_mouse    KERERQAKLAEEKMRKEEEEAKKRAEDDAKKKKVLSNMGAHFGGYLVKAEQKRGKRQTGR   167
               ****************:*******************************************
                                          Exon 10

TNNT1_dog      EMKLRILSERKKPLNIDHMGEDQLREKAQELSDWIHQLESEKFDLMEKLKQQKYEINVLY   239
TNNT1_sheep    EMKVRILSERKKPLNIDHMGEEQLREKAQELSDWIHQLESEKFDLMAKLKQQKYEINVLY   240
TNNT1_human    EMKVRILSERKKPLDIDYMGEEQLREKAQELSDWIHQLESEKFDLMAKLKQQKYEINVLY   239
Tnnt1_rat      EMKLRILSERKKPLNIDYMGEDQLREKAQELSEWIHQLESEKFDLMEKLKQQKYEINVLY   227
TNNT1_cow      EMKLRILSERKKPLNIDHMGEEQLREKAQELSDWIHQLESEKFDLMAKLKQQKYEINVLY   240
Tnnt1_mouse    EMKLRILSERKKPLNIDYMGEDQLREKAQELSEWIHQLESEKFDLMEKLKQQKYEINVLY   227
               ***:**********:**:***:*********:************* **************
                  Exon 11                          Exon 12

TNNT1_dog      NRISHAQKFRKGAGKGRVGGRWK       262
TNNT1_sheep    NRISHDQKFRKGAGKGRVGGRWK       263
TNNT1_human    NRISHAQKFRKGAGKGRVGGRWK       262
Tnnt1_rat      NRISHAQKFRKGAGKGRVGGRWK       250
TNNT1_cow      NRISHAQKFRKGAGKGRVGGRWK       263
Tnnt1_mouse    NRISHAQKFRKGAGKGRVGGRWK       250
               ***** *****************
               Exon 13      Exon 14
```
